# Serum uric acid levels are associated with obesity but not cardio-cerebrovascular events in Chinese inpatients with type 2 diabetes

**DOI:** 10.1038/srep40009

**Published:** 2017-01-04

**Authors:** Ming-Yun Chen, Cui-Chun Zhao, Ting-Ting Li, Yue Zhu, Tian-Pei Yu, Yu-Qian Bao, Lian-Xi Li, Wei-Ping Jia

**Affiliations:** 1 Department of Endocrinology and Metabolism, Shanghai Jiao Tong University Affiliated Sixth People’s Hospital; Shanghai Clinical Center for Diabetes; Shanghai Diabetes Institute; Shanghai Key Laboratory. of Diabetes Mellitus; Shanghai Key Clinical Center for Metabolic Disease; 600 Yishan Road, Shanghai 200233, China; 2 Department of VIP, Shanghai Jiao Tong University Affiliated Sixth People’s Hospital; 600 Yishan Road, Shanghai 200233, China

## Abstract

We aim to explore the associations between serum uric acid (SUA) and obesity and cardio-cerebrovascular events (CCEs) in Chinese inpatients with type 2 diabetes mellitus (T2DM). 2 962 inpatients with T2DM were stratified into quartile based on SUA concentrations. There were significant increases in the prevalence of both obesity (32.6%, 41.9%, 50.1%, and 62.8%, respectively, p < 0.001 for trend) and severe obesity (0.4%, 0.6%, 0.8%, and 1.3%, respectively, p < 0.001 for trend) across the SUA quartiles. A fully adjusted multiple logistic regression analysis revealed that SUA quartiles were independently associated with the presence of obesity (p < 0.001). The prevalence of CCEs was significantly higher in the obese diabetics than in the nonobese diabetics (16.8% vs. 13.2%, p = 0.027). After controlling for multiple confounding factors, BMI levels were also significantly correlated with the presence of CCEs (p = 0.020). However, there was no significant association of SUA quartiles/SUA levels with the presence of CCEs in T2DM. This study suggested that SUA levels were independently associated with obesity but not with CCEs in patients with T2DM. In selected populations such as subjects with T2DM, the role of uric acid in cardiovascular complications might be attributable to other cardiovascular risk factors, such as obesity.

In recent decades, the epidemic of hyperuricemia, obesity, cardiovascular and cerebrovascular diseases, which have cast heavy health and economic burden to the world^1^,^2^, have attained increasing recognition. Epidemiological and clinical evidence supports the strong link between serum uric acid (SUA) and obesity in different populations. For example, Dai *et al*.^3^ performed a cross-sectional study, including 27 009 middle- and old-age Chinese subjects, observed that BMI significantly increased with increasing SUA quintiles.

On the other hand, increasing evidence also demonstrates the tight association between obesity and cardio-cerebrovascular diseases in different populations^4^,^5^,^6^,^7^. For example, a collaborative analysis of 58 prospective studies, totally including 1.87 million person-years in risk, indicated that with BMI, the hazard ratio (HR) for cardiovascular disease (CVD) was 1.07 after further controlling age, sex, smoking status, blood pressure, total and HDL cholesterol and history of diabetes mellitus^7^.

However, the true relationships between SUA and atherosclerosis and cardiovascular complications still remain controversial. Although quantities studies have reported the positive association between SUA and cardiovascular diseases and mortality, it is still unclear whether SUA represents an independent causal risk factor or just confounded by other risk factors^8^,^9^. Several epidemiological studies have tried to elucidate if SUA is an independent risk factor for ischemic stroke, total cardiovascular diseases and cardiovascular mortality in general and diabetic populations^10^,^11^,^12^,^13^. For example, an earlier cohort study based on 83 683 Austrian men indicated that SUA was an independent predictor of cardiovascular mortality^13^. Also for stroke, compared to the lowest quintile of SUA, adjusted HR for the highest SUA quintile was 1.59^13^. However, substantial uncertainties on the importance of SUA in the prediction and evaluation of cardio-cerebrovascular risk remain, because uric acid is also strongly linked to other cardiovascular risk factors such as obesity, hypertension, diabetes mellitus, and metabolic syndromes, which may contribute to increased cardiovascular risk and mortality. Therefore, the observed associations of increased SUA concentrations with cardiovascular risk might be the consequences of confounding effects and merely a marker of risk for cardio-cerebrovascular diseases. For example, the British Regional Heart Study, a large prospective study including 7 735 men, showed that SUA was positively associated with risk for major coronary heart disease events. However, after fully adjusting for lifestyle factors and disease indicators, the causally independent relationship between SUA and major coronary heart disease events was no longer significant^14^.

Furthermore, a variety of studies have examined the relationship between SUA and cardiovascular risk in different populations, but few studies have focused on the associations between SUA and obesity and cardio-cerebrovascular events (CCEs) in Chinese patients with Type 2 diabetes mellitus (T2DM). Therefore, the aims of this study were to examine the associations between SUA and obesity and CCEs in hospitalized Chinese patients with type 2 diabetes.

## Methods

### Subjects and study design

The study was carried out in accordance with the “Declaration of Helsinki” and approved by the human research ethic committee of Shanghai Jiao Tong University Affiliated Sixth People’s Hospital, and all patients had signed written informed consent. This cross-sectional study was partly based on data used in our previous study^15^,^16^,^17^,^18^. Briefly, 3 598 inpatients with type 2 diabetes mellitus were consecutively observed. The patients who had taken any drug that might affect uric acid metabolism, such as losartan, furosemide and allopurinol were excluded. Finally, 2 962 patients including 1 676 men and 1 286 women were enrolled in our study. According to the SUA quartiles, the patients were divided into four groups. All patients were interviewed to obtain their history of hypertension (HTN), cardio-cerebrovascular events (CCEs), medicine use including metformin, lipid-lowering drugs (LLDs), antihypertensive agents (AHAs) and insulin or insulin analogues (IIAs), alcohol consumption and smoking habits. The definitions of smoking, alcohol consumption, and HTN were based on our previous criteria^15^,^16^. The definition of CCEs was also according to our previous study^15^,^18^. In brief, CCEs was defined as a history of transient ischemic attack, ischemic stroke, hemorrhagic stroke, angina, myocardial infarction, angioplasty, or coronary artery bypass surgery.

### Physical examinations and laboratory measurements

The physical examinations and laboratory measurements were carried out according to our previous studies^15^,^19^,^20^. Physical examinations such as weight, height, waist circumference, hip circumference and blood pressure were conducted following standardized protocol. The waist-to-hip ratio (WHR) was defined as waist circumference divided by hip circumference. Body mass index (BMI) was defined as weight divided by height^2^ (kg/m^2^). Underweight was defined as a BMI below 18.5 kg/m^2^, overweight as a BMI between 23 to 24.9 kg/m^2^, and obesity as BMI above 25 kg/m^2^ based on the Asia-Pacific criteria set by WHO^17^. Mild obesity was defined as BMI 25–30 kg/m^2^, moderate obesity as BMI 30–35 kg/m^2^, and severe obesity as BMI above 35 kg/m^2^ 21.

Blood samples were obtained to measure SUA, fasting plasma glucose(FPG), 2-h postprandial plasma glucose (2 h PPG), fasting C-peptide(FCP), 2-h postprandial C-peptide (2 h PCP), glycosylated hemoglobin A1c (HbA1c), total triglycerides (TTG), total cholesterol (TC), high-density lipoprotein cholesterol (HDL-C), low-density lipoprotein cholesterol (LDL-C), alanine aminotransferase (ALT), creatinine (Cr), C-reactive protein (CRP). The 24-h urinary albumin excretion (UAE) was measured as average of three separate early morning urine samples during hospitalization. The estimated glomerular filtration rate (eGFR) was calculated by equation for Chinese individuals: eGFR = 175 × (serum creatinine)^−1.234^ × (age)^−0.179^ (×0.79 if female)^20^.

### Statistical analysis

All data was analysed by SPSS 15.0. Normally distributed data was expressed as mean ± SD, whereas non-normally distributed data as medians or percentages (interquartile range 25–75%). For normal distributed variables, one-way ANOVA with LSD was performed to determine differences among groups. And kruskal-Wallis test was used for non-normal distributed variables. The chi-square test was used to compare the prevalence data and frequency differences. The partial correlation was used to determine the relationship between SUA and BMI. The binary logistic regression was applied to access the association of SUA/BMI with CCEs and the association between SUA quartiles and obesity. P < 0.05 was considered statistically significant.

## Results

### Clinical and laboratory data of the patients

The clinical characteristics of the patients according to SUA quartiles are shown in Table 1. Patients were stratified into quartiles based on SUA concentrations with the cutoff limits of <257, 257–311, 312–374, >374 umol/l. The patients with higher SUA quartiles were more likely to be men as expected. After adjustment for age and sex, prevalence of HTN, rate of use of metformin and IIAs and LLD and AHAs, SBP, DBP, BMI, WHR, FCP, 2 h PCP, TTG, TC, ALT, Cr and 24 h UAE progressively increased across the SUA quartiles (all p < 0.05). FPG, 2 h PPG, HbA1c, HDL-C and eGFR progressively decreased from the lowest SUA quartile to the highest quartile (all p < 0.05).

### Comparison of obesity between the SUA quartile groups

**Figure 1:**
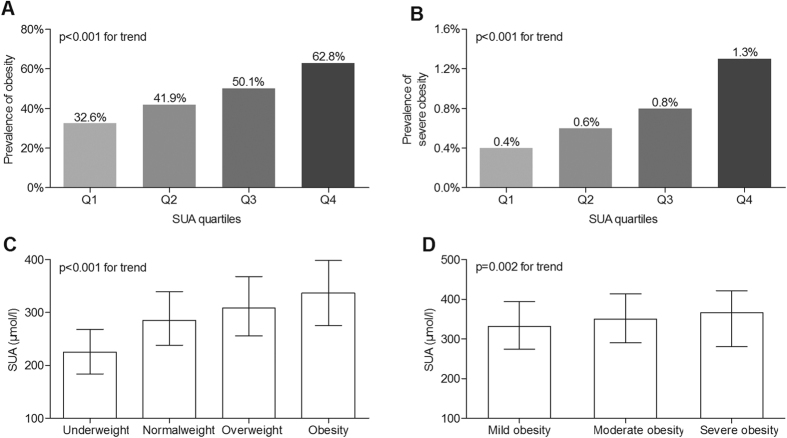
Comparison of obesity between the SUA quartile groups. Underweight was defined as a BMI below 18.5 kg/m^2^, overweight as a BMI between 23 to 24.9 kg/m^2^, and obesity as BMI above 25 kg/m^2^. Mild obesity was defined as BMI 25–30 kg/m^2^, moderate obesity as BMI 30–35 kg/m^2^, and severe obesity as BMI above 35 kg/m^2^. (**A**) Comparison of the prevalence of obesity among the four SUA quartile groups after controlling for age, sex and duration of diabetes. (**B**) Comparison of the prevalence of severe obesity among the four SUA quartile groups after controlling for age, sex and DD. (**C**) Comparison of SUA levels according to BMI after adjusting for age, sex and DD. (**D**) Comparison of SUA levels according to obesity degree after adjusting for age, sex and DD.

Figure 1 shows the comparison of obesity between SUA quartiles groups after controlling for age, sex and diabetic duration (DD). The prevalence of obesity significantly increased with the increasing SUA quartile (32.6%, 41.9%, 50.1% and 62.8% for the first, second, third and fourth quartiles, respectively, p < 0.001 for trend) (Fig. 1A). Moreover, there was also a significantly increasing trend in the prevalence of severe obesity across the SUA quartiles (0.4%, 0.6%, 0.8% and 1.3%, respectively, p < 0.001 for trend) (Fig. 1B). Furthermore, the SUA concentrations markedly increased with rising BMI among underweight, normalweight, overweight and obesity groups (p < 0.001 for trend) (Fig. 1C). Interestingly, the patients with more severe obesity tended to have higher SUA concentrations (p = 0.002 for trend) (Fig. 1D).

### Correlation between SUA and BMI

**Figure 2:**
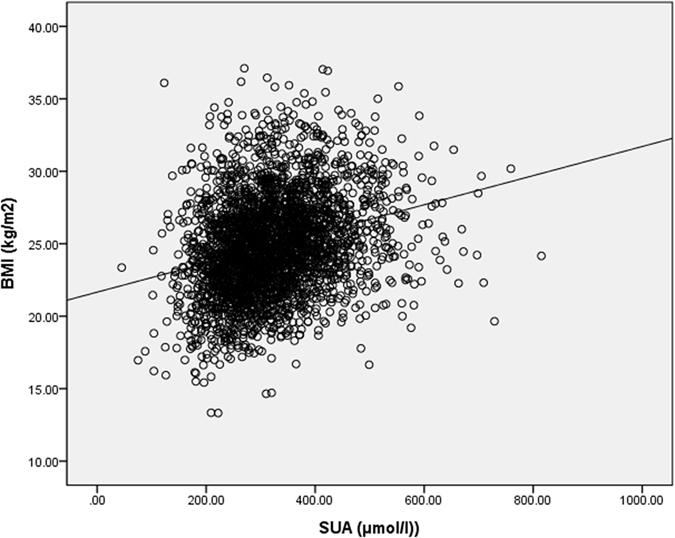
Correlation between SUA levels and BMI. R = 0.278, P < 0.001.

Figure 2 presents the correlation between SUA and BMI in all patients with T2DM. After adjusting for age, sex, and DD, the partial correlation analysis demonstrated the strongly positive correlation between SUA and BMI (R = 0.278, p < 0.001).

### Comparison of CCEs

**Figure 3:**
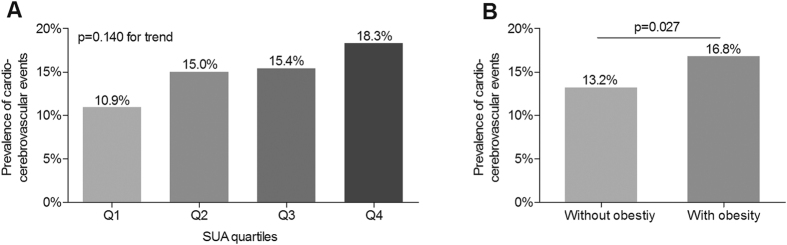
Comparison of CCEs. Obesity was defined as BMI above 25 kg/m^2^. (**A**) Comparison of the prevalence of cardio-cerebrovascular events among the four SUA quartile groups after controlling for age, sex and DD, HTN, smoking, and alcohol drinking. (**B**) Comparison of the prevalence of cardio-cerebrovascular events between the patients with and without obesity after controlling for age, sex and DD, HTN, smoking, and alcohol drinking.

Figure 3 demonstrates the comparison of CCEs in different groups. The prevalence of CCEs increased with the increasing SUA quartiles (10.9%, 15.0%, 15.4% and 18.3%, respectively), but no significant difference was observed after adjustment for age, sex, and DD (p = 0.140 for trend) (Fig. 3A). However, the prevalence of CCEs in the patients with obesity was significantly higher than those without obesity even controlling for age, sex, and DD (16.8% vs. 13.2%, p = 0.027) (Fig. 3B).

### Association of SUA quartiles with obesity

Table 2 presents the association of SUA quartiles with obesity in all patients with T2DM. After adjustment for age, sex, DD, HTN, smoking, and alcohol drinking (model1), the SUA quartiles were independently associated with an increased prevalence of obesity (p < 0.001 for trend). After additional adjustments for SBP, DBP, and the use of LLDs and AHAs and IIAs and metformin (model 2) and for ALT, TC, TTG, LDL-C, HDL-C, CRP, HbA1C, FPG, 2 h PPG, FCP, 2 h PCP, Cr, UAE, and eGFR (model 3), the SUA quartiles were still independently correlated with the increased prevalence of obesity (p < 0.001 for model 2 and 3). Compared with the lowest SUA quartile, the OR for obesity in the highest SUA quartile was 1.821 (95%CI, 1.373–2.414) after controlling multiple confounding factors (model 3).

### Association of SUA/BMI with CCEs

Table 3 displays the associations of SUA/BMI levels with CCEs. After controlling for multiple confounding factors, we failed to observe an independent association of SUA quartiles with the prevalence of CCEs (p > 0.05 for all models). However, similar to the association between SUA quartiles and obesity, BMI was also independently associated with the prevalence of CCEs (p < 0.001 for model 1, p = 0.013 for model 2, p = 0.012 for model 3, and p = 0.020 for model 4, respectively) after controlling multiple confounding factors.

## Discussion

Nowadays, hyperuricemia, obesity and atherosclerosis and its complications, such as cardiovascular diseases, have aroused extensive concern for public health in the international community because of their high prevalence worldwide, serious health consequences and substantial economic burden. Many epidemiological studies have tried to reveal the complex associations among them. However, substantial uncertainties still remain. Therefore, we conducted this hospital-based cross-sectional study to explore the associations between SUA and obesity and CCEs. Results of this study confirmed the strong association between SUA and obesity, and also showed the strong relationship between obesity and CCEs. However, our study observed the absence of a relationship between SUA and CCEs after adjustment for other risk factors especially obesity in Chinese patients with type 2 diabetes.

We observed that the prevalence of obesity steadily increased across SUA quartiles, and SUA levels tightly and independently related to obesity in T2DM even after controlling for other obesity risk factor, such as age and use of insulin. In a recent study, we also found that both BMI and WHR significantly increased with the increase of SUA concentrations in T2DM^19^. Consistent with our results, several epidemiological studies have also shown positive relationship between SUA and obesity^22^,^23^,^24^. For example, in a 10-year follow-up study, Rathmann *et al*. found that the BMI in all race-sex-groups significantly increased with increasing uric acid level^25^. Furthermore, not only elevated SUA concentrations were associated with the increasing risk of obesity, but obesity was also associated with higher risk of hyperuricemia. For example, Tanaka *et al*.^26^ found that BMI was significantly associated with SUA in Japanese adult twins, after adjusting for both genetic and familial environment factors. Interestingly, a prospective longitudinal study including 60 subjects with T2DM and severe obesity (BMI > 35 kg/m^2^) reported the significant reduction in SUA concentrations (p = 0.0002 compared with baseline) after bariatric surgery, which led to an average 34.3 kg weight reduction, while no change in SUA after non-surgical weight loss of more than 5 kg^27^. Therefore, weight-loss surgery may be an efficient therapy to lower SUA concentrations in the subjects with obesity, especially with severe obesity.

In addition to strong association with SUA, obesity is also closely associated with a number of other conditions such as diabetes mellitus, dyslipidaemia, atherosclerosis, and cardiovascular complications^28^,^29^,^30^,^31^,^32^. Among these disorders, atherosclerosis and its complications such as coronary artery disease and stroke are among the leading cause of morbidity and mortality in type 2 diabetes. In general, obesity is thought to exert detrimental effects on the cardio-cerebrovascular system, which lead to increased cardiovascular and cerebrovascular risks in T2DM. Therefore, in the present study, we also found obesity strongly associated with CCEs based on multiple regression analysis. In consistence with our study, multiple clinical and epidemiological studies reported the clear and strong link between obesity and CCEs in different populations^28^,^29^,^30^,^31^,^32^. For example, The 45 and Up Study, a large-scale Australian cohort study, investigators analyzed the correlation between BMI and incident hospitalization for cardiovascular disease among 158 546 participants, and confirmed that with increasing BMI, the risk of hospitalization for cardiovascular disease subtypes such as angina and acute myocardial infarction were significantly increased^31^. Moreover, obesity not only links to CVD events, but also relates to cerebrovascular events. For example, Strazzullo *et al*.^32^ performed a meta-analysis including 25 prospective studies to explore the association between overweight, obesity and incidence of stroke. They demonstrated that the association between BMI and stroke mortality was J-shaped, and that both overweight and obesity are significantly related to progressively increasing risk of ischemic stroke independent of other cardiovascular risk factors^32^.

Although the markedly positive association between SUA and obesity, and also between obesity and CCEs were observed, we failed to find the direct relationship between SUA and CCEs after adjustment for age, sex, and DD. The present findings accord with our recent studies^19^,^20^,^33^. These studies showed that increased SUA concentrations were closely associated with HTN, metabolic syndrome, and decreased urine uric acid excretion (UUAE) was significantly related to both diabetic retinopathy and chronic kidney disease in type 2 diabetes^19^,^20^,^33^. Furthermore, HTN, metabolic syndrome, diabetic retinopathy and chronic kidney disease were closed associated with atherosclerosis in patients with type 2 diabetes^19^,^20^,^33^. Currently, it is accepted that HTN, metabolic syndrome, diabetic retinopathy and chronic kidney disease were important risk factors for atherosclerosis and its cardiovascular complications. However, both SUA and UUAE levels were not associated with carotid and lower limb atherosclerosis in type 2 diabetes^19^,^20^,^33^. Therefore, our recent studies further indicate that the role of uric acid in atherosclerosis and cardiovascular diseases is indirect and may result from other cardiovascular risk factors such as HTN, chronic kidney disease and obesity.

Presently, the results regarding the true relationship between SUA and atherosclerosis and its complications such CCEs have been conflicting in different studies. Although several studies found that elevated SUA was an independent risk factor for independently predicted CCEs^34^,^35^,^36^, some other studies have reported that SUA is not an independent risk factor for CCEs in different population including diabetic population^14^,^37^,^38^. Furthermore, there have also been convicting studies argued that elevated SUA concentration is merely a risk marker for CCEs, and the correlation between SUA and CCEs is equally poor^39^,^40^. Also, The Framingham Heart Study, a follow-up of 117 376 person-years, reported that in stepwise Cox models, after additional adjustment for risk factors, the correlation between SUA and cardiovascular events diminished, and diuretic use was identified as the covariate factor^37^. In addition, Panero *et al*.^40^ confirmed that there was no significant association between SUA and cardiovascular mortality. Instead, SUA was closely related to the metabolic syndrome components including hypertension, obesity and dyslipidemia, which provided higher cardiovascular risk. It is well known that hypertension, obesity and dyslipidemia are traditional cardiovascular risk factors, which lead to increased morbidity and mortality of cardiovascular diseases. Furthermore, Palmer *et al*.^38^ analyzed variation at uric acid-related genes SLC2A9 and BMI-related gene including FTO, MC4R, and TMEM18, and indicated that there was no evidence to support the causal link between SUA and ischaemic heart disease.

Given the absence of association between SUA and CCEs, and the presence of association of obesity with both SUA and CCEs, we speculated that in selected populations, such as those with T2DM, the association of SUA with atherosclerosis and its complications may be indirect and mediated by other cardiovascular risk factors such as obesity, diabetic retinopathy and chronic kidney disease. Our recent studies also support this view^19^,^20^,^33^, in which both SUA and UUAE are closely associated with cardiovascular risk factors including HTN, metabolic syndrome, diabetic retinopathy and chronic kidney disease, but not with atherosclerosis in T2DM. Also, Viazzi *et al*.^41^ reported uric acid is a reliable predictor of blood pressure over time in Children at Cardiovascular Risk. And a study based on Chinese community-dwelling population reported that SUA are significantly associated with BMI, blood pressure and dyslipidemia, which are cardiometabolic risk factors^42^. Among these cardiovascular risk factors, for example, HTN plays an important role in the formation and progression of atherosclerotic lesions and its cardiovascular complications, and has been recognized as one of the most risk factors for cardiovascular and cerebrovascular diseases. Therefore, these cardiovascular risk factors might be chains that link uric acid with atherosclerosis and cardiovascular risk in T2DM. The true role of uric acid in atherosclerosis and its complications is ambiguous and need further clarification.

There are several limitations should be noted. First of all, the cross-sectional nature of the data cannot prove cause-effect relationships between SUA and obesity and CCEs. We therefore could not assess the temporal association between SUA and CCEs risk factors. In addition, all participants were Chinese inpatients with T2DM, the conclusion of our study may not apply to other ethnic populations. Furthermore, the mechanism underlying the true relationship between SUA and obesity and CCEs still remain to explore.

In conclusion, our findings suggest that SUA is closely related to obesity but not to CCEs in Chinese patients with T2DM. Contrary to SUA, obesity is an independent risk factor for CCEs in patients with T2DM. In selected populations, such as those with T2DM, the association between SUA and CCEs might be attributable to other cardiovascular risk factors, such as obesity. Prospective studies are required to clarify the causal associations of SUA with obesity and CCEs in T2DM.

## Additional Information

**How to cite this article**: Chen, M.-Y. *et al*. Serum uric acid levels are associated with obesity but not cardio-cerebrovascular events in Chinese inpatients with type 2 diabetes. *Sci. Rep.* **7**, 40009; doi: 10.1038/srep40009 (2017).

**Publisher's note:** Springer Nature remains neutral with regard to jurisdictional claims in published maps and institutional affiliations.

## Figures and Tables

**Table 1:** Characteristics of the subjects according to SUA quartiles.

Variables	Q1 (n = 733)	Q2 (n = 742)	Q3 (n = 740)	Q4 (n = 747)	p value	*p value
SUA (umol/l)	<257	257–311	312–374	>374	—	—
Age (years)	60 ± 12	59 ± 12	59 ± 13	59 ± 13	0.924	0.262
Male (n, %)	294 (40.1%)	392 (52.8%)	458 (61.9%)	532 (71.2%)	<0.001	<0.001
*DD (months)	72 (12–132)	84 (24–144)	72 (24–132)	72 (24–132)	0.314	0.198
Smoking (n, %)	161 (22.0%)	219 (29.5%)	218 (29.5%)	254 (34.0%)	<0.001	0.264
Alcohol (n, %)	77 (10.5%)	113 (15.2%)	123 (16.6%)	148 (19.8%)	<0.001	0.785
HTN (n, %)	308 (42.0%)	368 (49.6%)	414 (55.9%)	488 (65.3%)	<0.001	<0.001
Metformin (n, %)	367 (50.1%)	409 (55.1%)	427 (57.7%)	432 (57.8%)	0.003	<0.001
IIAs (n, %)	556 (75.9%)	546 (73.6%)	475 (64.2%)	502 (67.2%)	<0.001	<0.001
LLD (n, %)	163 (22.2%)	213 (28.7%)	231 (31.2%)	306 (41.0%)	<0.001	<0.001
AHAs (n, %)	276 (37.7%)	339 (45.7%)	374 (50.5%)	453 (60.6%)	<0.001	<0.001
SBP (mmHg)	130 ± 17	131 ± 18	133 ± 17	134 ± 17	<0.001	<0.001
DBP (mmHg)	79 ± 10	79 ± 9	81 ± 9	81 ± 10	<0.001	<0.001
BMI (kg/m2)	23.68 ± 3.47	24.49 ± 3.18	25.22 ± 3.31	26.18 ± 3.26	<0.001	<0.001
WHR	0.89 ± 0.07	0.91 ± 0.06	0.92 ± 0.06	0.93 ± 0.06	<0.001	<0.001
*FPG (mmol/l)	8 (6.3–10.3)	7.9 (6.3–9.7)	7.6 (6.1–9.3)	7.4 (6.1–9.3)	<0.001	<0.001
*2 h PPG (mmol/l)	14.4 (10.6–17.5)	13.5 (9.8–16.9)	13.2 (10–16.2)	13.2 (9.8–16.8)	0.008	0.048
HbA1C (%)	9.8 ± 2.51	9.25 ± 2.37	8.81 ± 2.4	8.6 ± 2.18	<0.001	<0.001
*FCP (ng/mL)	1.3 (0.77–1.9)	1.52 (0.88–2.32)	1.82 (1.18–2.52)	2.13 (1.43–3.09)	<0.001	<0.001
*2h C-P (ng/mL)	2.71 (1.54–4.45)	3.35 (1.85–5.06)	4.18 (2.43–5.62)	4.67 (2.94–5.8)	<0.001	<0.001
*TG (mmol/l)	1.18 (0.84–1.65)	1.34 (0.95–1.96)	1.46 (1.01–2.16)	1.86 (1.28–2.76)	<0.001	<0.001
TC (mmol/l)	4.65 ± 1.12	4.68 ± 1.16	4.67 ± 1.05	4.83 ± 1.2	0.009	<0.001
HDL ± C (mmol/l)	1.21 ± 0.32	1.15 ± 0.33	1.07 ± 0.26	1.02 ± 0.26	<0.001	<0.001
LDL ± C (mmol/l)	3.09 ± 0.94	3.1 ± 0.99	3.07 ± 0.89	3.13 ± 0.97	0.642	0.142
*ALT (U/l)	17 (12–26)	19 (13–28)	19.5 (14–31)	22 (15–36)	<0.001	<0.001
*Cr (μmol/l)	57 (49–68)	64 (55–76)	69 (59–81)	78 (66–96)	<0.001	<0.001
*24 h UAE (mg)	10 (6–22)	10 (6–21)	11 (6–29)	15 (7–65)	<0.001	<0.001
*eGFR (ml/min/1.73 m^2^)	125 (103–148)	111 (96–133)	106 (89–127)	93 (72–115)	<0.001	<0.001
*CRP (mg/l)	0.96 (0.37–2.57)	1.05 (0.44–2.49)	1.15 (0.49–2.77)	1.4 (0.67–3.55)	<0.001	0.106

Values are expressed as the mean ± S.D, or median with interquartile range, or percentages.

*Non-normal distribution of continuous variables.

P-value: The p-values were not adjusted for sex for the trend.

*P-value: The *p-values were adjusted for sex for the trend.

Abbreviations: DD, diabetic duration; HTN, hypertension; LLDs, lipid-lowering drugs; AHAs, antihypertensive agents; IIAs, insulin or insulin analogues; UAE, urinary albumin excretion.

**Table 2:** Association of SUA quartiles with obesity.

	ORs (95% CI)				*P* values for trend
	Q1	Q2	Q3	Q4	
Model 1	1	1.451 (1.167–1.805)	2.060 (1.652–2.569)	3.318 (2.642–4.166)	<0.001
Model 2	1	1.384 (1.106–1.733)	1.870 (1.490–2.347)	2.978 (2.355–3.765)	<0.001
Model 3	1	1.217 (0.932–1.590)	1.477 (1.130–1.930)	1.821 (1.373–2.414)	<0.001

Model 1: adjusted for age, sex, DD, HTN, smoking, and alcohol drinking.

Model 2: further adjusted for SBP, DBP, and the use of LLDs and AHAs and IIAs and metformin.

Model 3: further adjusted for ALT, TC, TTG, LDL-C, HDL-C, CRP, HbA1C, FPG, 2 h PPG, FCP, 2 h PCP, Cr, UAE, and eGFR.

Abbreviations: DD, diabetic duration; HTN, hypertension; LLDs, lipid-lowering drugs; AHAs, antihypertensive agents; IIAs, insulin or insulin analogues; UAE, urinary albumin excretion.

**Table 3:** Association of SUA/BMI with cardio-cerebrovascular events.

	ORs	95% CI	P values
**Model 1**			
SUA	1.001	1.000–1.002	0.059
BMI	1.080	1.045–1.115	<0.001
**Model 2**			
SUA	1.000	0.999–1.001	0.793
BMI	1.044	1.009–1.080	0.013
**Model 3**			
SUA	1.000	0.999–1.001	0.987
BMI	1.046	1.010–1.084	0.012
**Model 4**			
SUA	0.999	0.997–1.001	0.419
BMI	1.055	1.009–1.103	0.020

Model 1 adjusted for age, gender, duration of diabetes, SUA and BMI.

Model 2 further adjusted for HTN, smoking, alcohol drinking, and the use of LLDs and AHAs and IIAs and metformin.

Model 3 further adjusted for SBP, DBP, and WHR.

Model 4 further adjusted for ALT, TC, TTG, LDL-C, HDL-C, Cr, eGFR, UAE, CRP, HbA1C, FCP, 2 h CP,

FPG, and 2 h PPG.

Abbreviations: HTN, hypertension; LLDs, lipid-lowering drugs; AHAs, antihypertensive agents; IIAs, insulin or insulin analogues; UAE, urinary albumin excretion.
